# Construction of new solutions of Korteweg-de Vries Caudrey-Dodd-Gibbon equation using two efficient integration methods

**DOI:** 10.1371/journal.pone.0275118

**Published:** 2022-09-27

**Authors:** Saima Arshed, Ghazala Akram, Maasoomah Sadaf, Komal Saeed

**Affiliations:** Department of Mathematics, University of the Punjab, Quaid-e-Azam Campus, Lahore, Pakistan; China University of Mining and Technology, CHINA

## Abstract

Korteweg-de Vries Caudrey-Dodd-Gibbon (KdV-CDG) equation describes many physical phenomena in plasma physics, optical fibers, dynamics of the ocean, quantum mechanics, acoustic waves and laser optical applications. In this paper, the KdV-CDG equation is analyzed via two reliable and efficient integrating approaches. The suggested techniques; the extended $\frac{G^{\prime }}{G^{2}}$-expansion method and exponential (*ψ*(*ξ*))-expansion method successfully extract hyperbolic function solutions, trigonometric function solutions and rational function solutions. The existence criteria for all the obtained solutions are also discussed in this paper. At the end, various 3D and 2D contour plots have been constructed for better understanding of constructed solutions.

## Introduction

Nonlinear partial differential equations (NLPDEs) are used to investigate and model many physical problems occurring in the real-world. NLPDEs have immense applications in all sectors of life and have attracted the attention of researchers and scientists. It is observed that many of the NLPDEs are reported to have the exact solutions in the form of traveling wave functions, often depicting solitary waves or solitons.

Solitons were first discovered by Scott Russell in 1834. Since then, many scientists have contributed toward understanding solitons and their impact on real world applications. Solitons act like solitary waves which hold the law of conservation. Solitary waves have both particle-like and wave-like natures. Upon collision with other waves, soliton waves conserve their shape. Solitons occur due to the balance between two effects known as dispersive and nonlinear effects. These properties have motivated the researchers to find exact solutions to NLPDEs. Solitons have numerous applications in telecommunication, acoustics, optical fiber, fluid mechanics and in various sectors of physics. Much research has been done to find soliton solutions of many nonlinear partial differential equations as they link mathematics and physics together. In this regard major work has been done which includes the following studies. The construction of solitary waves, breather waves and hybrid waves for (3 + 1)-dimensional NLEE is studied in [1]. The higher-order nonlinear Schrödinger-Maxwell-Bloch equations are investigated in [2]. Stability analysis, solitary wave and explicit power series solutions of a (2 + 1)-dimensional nonlinear Schrödinger equation is discussed in [3]. The Riemann-Hilbert problem is developed to study the nonlinear Schrödinger equation in [4]. Moreover, the higher order NLSE is studied using Darboux-dressing transformation with the Lax pair and asymptotic expansion method in [5]. The nonlinear wave transitions of (2 + 1)-dimensional Sawada-Kotera is investigated in [6]. Many techniques are used to extract soliton solutions such as the generalized projective Riccati equation method [7], improved $\text{tan}(\frac{\psi (\eta )}{2})$ method [8], (*G*′/*G*, 1/*G*)-expansion approach [9] and $\bar{\partial }$-dressing method [10] and [11].

In this paper, the Korteweg-de Vries Caudery-Dodd-Gibbon equation is studied using two powerful and efficient analytical techniques such as extended $\frac{G^{\prime }}{G^{2}}$-expansion method and exponential (*ψ*(*ξ*))-expansion method. These methods have been found useful to solve many complex problems with impressive results. It has been observed that the extended $\frac{G^{\prime }}{G^{2}}$-expansion method has been efficiently used for investigating problems such as time-fractional Burgers equation, fractional biological population model, space-time fractional Whitham-Broer-Kaup equations [12] and Triki-Biswas equation [13]. The exponential (*ψ*(*ξ*))-expansion method is applied on Nizhnik-Novikov-Veselov model [14] and on strain wave equation [15] to extract the soliton solutions. This research deals with the extraction of exact solutions for KdV-CDG equation using the proposed techniques.

This study is organized as follows: In Section 2, the governing equation is explained. Section 3 contains detailed description of the proposed methods. Section 4 contains all newly constructed solutions extracted as a result of employing the suggested techniques. Section 5 contains the graphical representation of few of the specified solutions and in the last section, concluding remarks are presented.

## Korteweg-de Vries Caudrey-Dodd-Gibbon (KdV-CDG) equation

This study is based on investigating the combined form of Korteweg-de Vries and Caudrey-Dodd-Gibbon equations; termed as Korteweg-de Vries Caudrey-Dodd-Gibbon (KdV-CDG) equation. Both KdV and CDG equations have numerous applications in water waves. They play a vital role in nonlinear studies such as; plasma physics, dynamics of ocean, quantum mechanics, acoustic wave and in laser optics [16]. The KdV-CDG equation is given, as

$$\begin{array}{c} v_{t}+l(v_{xx}+\frac{1}{5}\beta v^{2}{)}_{x}+m(\frac{1}{15}\beta v^{3}+\beta vv_{xx}+v_{xxxx}{)}_{x}=0. \end{array}$$ (1)

If *m* = 0 in Eq (1), then it reduces to Korteweg-de Vries (KdV) equation. If *l* = 0 in Eq (1), then it reduces to Caudrey-Dodd-Gibbon (CDG). In 1877, Boussinesq introduced the KdV equation for the first time and then later major work on it was done by Korteweg and de Vries. In 1895, they developed this linear model to describe solitary waves. Small amplitude shallow water waves, surface waves of long wavelength and internal waves in a shallow density-stratified fluid are described by KdV equation [17, 18]. CDG is integrable model and provides various solutions with limited number of conserved quantities [19]. The unique properties and numerous applications of the KdV-CDG equation has opened new horizons for researchers. Recently, KdV-CDG model has been examined using different approaches including F-expansion method, extended hyperbolic function method and exponential function method [20–22].

## Description of methods

This section contains the description of the proposed methods.

The general nonlinear evolution equation is considered, as

$$\begin{array}{c} E(v,D_{t}v,D_{x}v,D_{t}^{2}v,D_{xt}v,D_{x}^{2}v,\ldots )=0, \end{array}$$ (2)

where *v* = *v*(*x*, *t*) is considered as an unknown function, *E* represents a polynomial function in *v*. The following traveling wave transformation is used to convert Eq (2) into an ODE, as

$$\begin{array}{c} v(x,t)=s(\xi ),\;\;\xi =x-\rho t, \end{array}$$

where *ρ* represents velocity of the wave profile. The transformed ODE has the form

$$\begin{array}{c} F(s,s^{{}^{\prime }},s^{{}^{\prime \prime }},s^{{}^{\prime \prime \prime }},\ldots )=0, \end{array}$$ (3)

where ′ indicates the derivative with respect to *ξ*.

### Method I: The exponential (*ψ*(*ξ*))-expansion method

According to **Method I**, the solution of ODE Eq (3) has the form,

$$\begin{array}{c} s\left(\xi \right)=\sum_{i=0}^{n}w_{i}{\left(\text{exp}\left(-\psi \left(\xi \right)\right)\right)}^{i}, \end{array}$$ (4)

where *w*_*i*_ represents the unknown constants that are determined later. *ψ*(*ξ*) obeys the relation

$$\begin{array}{c} \psi^{{}^{\prime }}\left(\xi \right)=\text{exp}\left(-\psi \left(\xi \right)\right)+\mu \;\text{exp}\left(\psi \left(\xi \right)\right)+\lambda . \end{array}$$ (5)

Eq (5) possesses the following three types of solutions.

**Case 1**: (λ^2^ − 4*μ* > 0 **and** *μ* ≠ 0)

Hyperbolic solution is obtained, as

$$\begin{array}{ccc} \psi_{1}\left(\xi \right) & = & \text{ln}(\frac{-\sqrt{\lambda^{2}-4\mu }\;\text{tanh}(\frac{\sqrt{\lambda^{2}-4\mu }}{2}\left(\xi +c\right))-\lambda }{2\mu }). \end{array}$$ (6)

**Case 2**: (λ^2^ − 4*μ* < 0 **and** *μ* ≠ 0)

Trigonometric solution is gained in Case 2, as

$$\begin{array}{ccc} \psi_{2}\left(\xi \right) & = & \text{ln}(\frac{\sqrt{4\mu -\lambda^{2}}\;\text{tan}\;(\frac{\sqrt{4\mu -\lambda^{2}}}{2}\left(\xi +c\right))-\lambda }{2\mu }). \end{array}$$ (7)

**Case 3**: (λ^2^ − 4*μ* > 0 **and** *μ* = 0 **and** λ ≠ 0)

In this case, hyperbolic function solution is obtained, as

$$\begin{array}{ccc} \psi_{3}\left(\xi \right) & = & -\text{ln}(\frac{\lambda }{\text{cosh}(\lambda (\xi +c))+\text{sinh}(\lambda (\xi +c))-1}). \end{array}$$ (8)

**Case 4**: (λ^2^ − 4*μ* = 0 **and** *μ* ≠ 0 **and** λ ≠ 0)

Rational solution is extracted in this case, as

$$\psi_{4}\left(\xi \right)=\text{ln}(-\frac{2(\lambda (\xi +c))+2}{\lambda^{2}\left(\xi +c\right)}).$$ (9)

**Case 5**: (λ^2^ − 4*μ* = 0 **and** *μ* = 0 **and** λ = 0)

Solutions will be of the form

$$\begin{array}{c} \psi_{5}\left(\xi \right)=\text{ln}\left(\xi +c\right), \end{array}$$ (10)

where *c* is a constant of integration.

Through homogenous balancing, the value of *n* is determined. Inserting Eq (4) into Eq (3) and using Eq (5), a system of equations in *w*_*i*_ is retrieved by computing the coefficients of every power of exp(-*ψ*(*ξ*)) to 0. Solving the obtained system gives the values of the unknown parameters.

### Method II: The extended ($\frac{G^{{}^{\prime }}}{G^{2}}$)-expansion method

According to **Method II**, the assumed solution of Eq (3) has the form

$$\begin{array}{c} s\left(\xi \right)=c_{0}+\sum_{i=1}^{n}[c_{i}(\frac{G^{{}^{\prime }}}{G^{2}}{)}^{i}+d_{i}(\frac{G^{{}^{\prime }}}{G^{2}}{)}^{-i}] \end{array}$$ (11)

where $\frac{G^{\prime }}{G^{2}}$ satisfies the ODE,

$$\begin{array}{c} (\frac{G^{{}^{\prime }}}{G^{2}}{)}^{{}^{\prime }}=\lambda +\mu (\frac{G^{{}^{\prime }}}{G^{2}}{)}^{2}. \end{array}$$ (12)

λ ≠ 1 and *μ* ≠ 0 are integers while *c*_0_, *c*_*i*_ and *d*_*i*_ (*i* = 1, 2, 3, …, *n*) are arbitrary parameters which have to be determined. The value of *n* is obtained by homogenous balancing.

**Step 2**

Putting Eqs (11) and (12) into Eq (3), an algebraic system of equations is obtained by equating the coefficients of different powers of $(\frac{G^{{}^{\prime }}}{G^{2}}{)}^{j}$, (*j* = 0, ±1, 2, …) to zero. Solving the obtained system gives the values of arbitrary parameters.

**Step 3**

Eq (12) has three different forms of solutions:

**Case 1**

Trigonometric form of solutions are obtained if λ*μ* > 0:

$$\begin{array}{ccc} \frac{G^{{}^{\prime }}}{G^{2}} & = & \sqrt{\frac{\lambda }{\mu }}[\frac{H_{1}\;\text{cos}\left(\sqrt{\mu \lambda }\xi \right)+H_{2}\;\text{sin}\left(\sqrt{\mu \lambda }\xi \right)}{H_{2}\;\text{cos}\left(\sqrt{\lambda \mu }\xi \right)-H_{1}\;\text{sin}\left(\sqrt{\lambda \mu }\xi \right)}]. \end{array}$$ (13)

**Case 2**

Hyperbolic form of solutions are obtained if λ*μ* < 0:

$$\begin{array}{ccc} \frac{G^{{}^{\prime }}}{G^{2}} & = & -\frac{\sqrt{|\lambda \mu }|}{\mu }[\frac{H_{1}\;\text{sinh}\left(2\sqrt{\left|\mu \lambda \right|}\xi \right)+H_{1}\;\text{cosh}\left(2\sqrt{\left|\mu \lambda \right|}\xi \right)+H_{2}}{H_{1}\;\text{sinh}\left(2\sqrt{\left|\mu \lambda \right|}\xi \right)+H_{1}\;\text{cosh}\left(2\sqrt{\left|\mu \lambda \right|}\xi \right)-H_{2}}]. \end{array}$$ (14)

**Case 3**

When λ = 0 and *μ* ≠ 0, then rational solutions are obtained, as

$$\begin{array}{c} \frac{G^{{}^{\prime }}}{G^{2}}=-\frac{H_{1}}{\mu (H_{1}\xi +H_{2})}. \end{array}$$ (15)

*H*_1_ and *H*_2_ are considered as arbitrary parameters.

**Step 4**

By substituting *c*_0_, *c*_*i*_, *d*_*i*_ and $\frac{G^{{}^{\prime }}}{G^{2}}$ in Eq (11), the solutions of Eq (3) are obtained.

## Mathematical analysis of KdV-CDG equation

This part of the paper is dedicated to applying the afore mentioned techniques on the KdV-CDG equation to draw out new soliton solutions. The obtained results may be found helpful in understanding the complex nonlinear phenomena arising in plasma physics, optical fibers, dynamics of the ocean, quantum mechanics and acoustic waves. For applying both methods, the following traveling wave transformation is employed, as

$$\begin{array}{c} v(x,t)=s(\xi ),\;\xi =x-\rho t. \end{array}$$

This transformation, converts Eq (1) into the following ODE.

$$\begin{array}{c} -\rho s^{{}^{\prime }}+l{\left(s^{{}^{\prime \prime }}+\frac{1}{5}\beta s^{2}\right)}^{{}^{\prime }}+m{\left(\frac{1}{15}\beta s^{3}+\beta s^{{}^{\prime \prime }}s+s^{{}^{\prime \prime \prime \prime }}\right)}^{{}^{\prime }}=0. \end{array}$$ (16)

After integrating Eq (16) and putting constant of integration to zero, the following equation is obtained.

$$\begin{array}{c} -\rho s+l\left(s^{{}^{\prime \prime }}+\frac{1}{5}\beta s^{2}\right)+m\left(\frac{1}{15}\beta s^{3}+\beta s^{{}^{\prime \prime }}s+s^{{}^{\prime \prime \prime \prime }}\right)=0. \end{array}$$ (17)

### Construction of soliton solutions via technique I

In this subsection, the technique I has been applied on the proposed model to obtain new soliton solutions. Application of homogenous balancing on Eq (17) yields *n* = 2. Inserting *n* = 2 in Eq (4), gives

$$\begin{array}{c} s\left(\xi \right)=w_{0}+w_{1}\;\text{exp}\;\psi \left(\xi \right)+w_{2}{\left(\text{exp}\;\psi \left(\xi \right)\right)}^{2}. \end{array}$$ (18)

Utilizing exponential (*ψ*(*ξ*))-expansion method, an algebraic system of equations is obtained with the following solution sets.

1^*st*^ **Solution set**:

$$\begin{array}{ccc} & & w_{0}=-\frac{\sqrt{3}\sqrt{3l^{2}-10lm(\lambda^{2}-4\mu )+35m^{2}(\lambda^{2}-4\mu {)}^{2}}+3l+15m(\lambda^{2}+4\mu )}{4m},\\ & & w_{1}=-30\lambda ,\;w_{2}=-30,\;\beta =1. \end{array}$$

In 1^*st*^ **Solution set** (λ^2^ − *μ*) is taken as arbitrary constant so all the cases have been considered which are as follows:

**Case 1**: λ^2^ − 4*μ* > 0, *μ* ≠ 0

Hyperbolic solution is obtained, as

$$\begin{array}{c} \begin{array}{cc} v_{1}\left(x,t\right)= & -\frac{\sqrt{3}\sqrt{3l^{2}-10lm(\lambda^{2}-4\mu )+35m^{2}{\left(\lambda^{2}-4\mu \right)}^{2}}+3l+15m(\lambda^{2}+4\mu )}{4m}\\ & +\frac{60\lambda \mu }{\sqrt{\lambda^{2}-4\mu }\;\text{tanh}\;(\frac{1}{2}\left(c+\xi \right)\sqrt{\lambda^{2}-4\mu })+\lambda }\\ & -\frac{120\mu^{2}}{{\left(\sqrt{\lambda^{2}-4\mu }\;\text{tanh}\;(\frac{1}{2}\left(c+\xi \right)\sqrt{\lambda^{2}-4\mu })+\lambda \right)}^{2}}. \end{array} \end{array}$$ (19)

**Fig 1:**
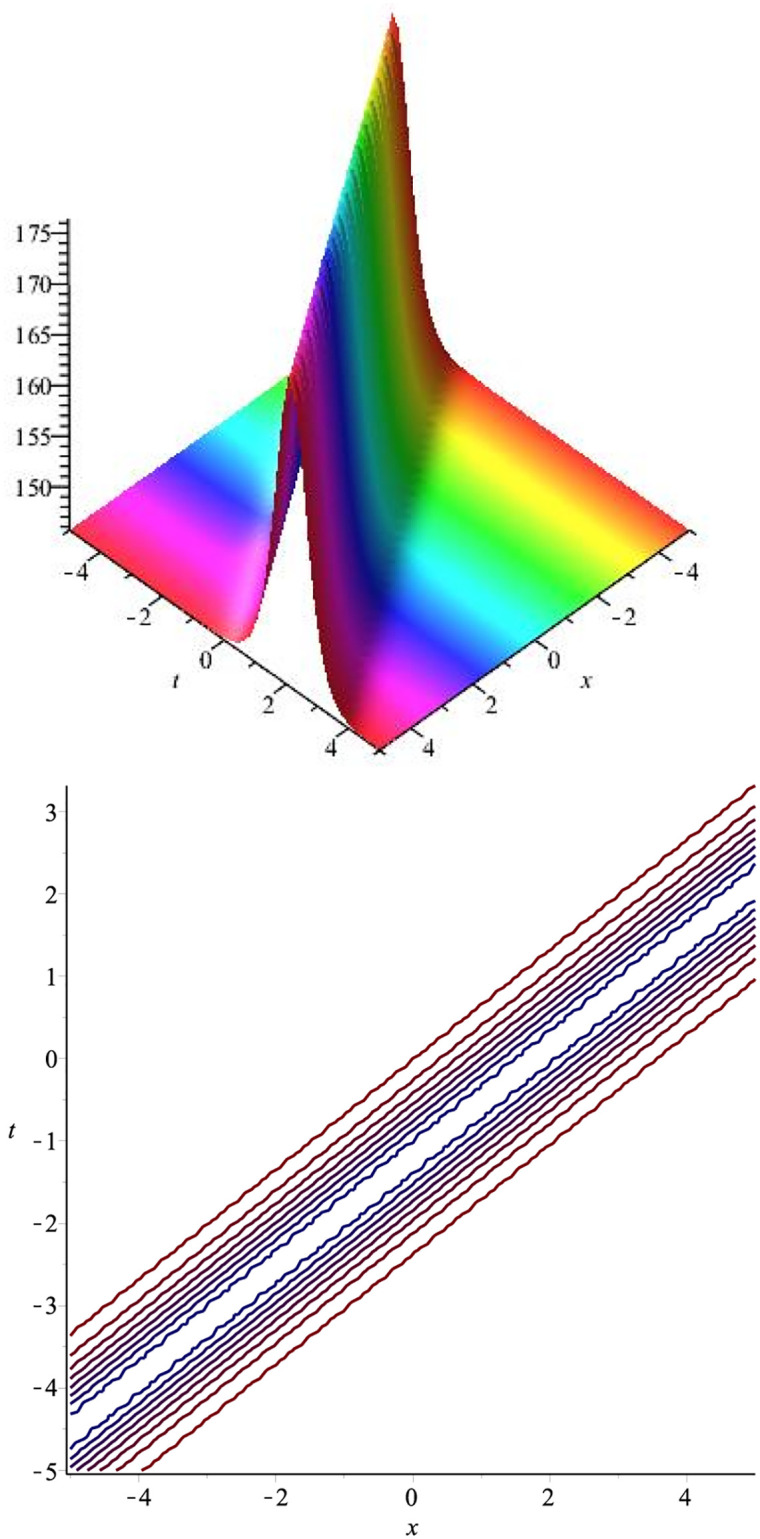
Bright soliton: ∣*v*_1_(*x*, *t*)∣ for *l* = 1.9, *m* = 1.5, λ = 3.7, *c* = −2.4, *v* = 1.5, *β* = 1, *μ* = 2.4.

The graphical representation of the above obtained solution is represented in Fig 1.

**Case 2**: λ^2^ − 4*μ* < 0, *μ* ≠ 0

Case 2 extracts trigonometric function solution, as

$$\begin{array}{c} \begin{array}{cc} v_{2}\left(x,t\right)= & -\frac{\sqrt{3}\sqrt{3l^{2}-10lm(\lambda^{2}-4\mu )+35m^{2}{\left(\lambda^{2}-4\mu \right)}^{2}}+3l+15m(\lambda^{2}+4\mu )}{4m}\\ & +\frac{60\lambda \mu }{\lambda -\sqrt{4\mu -\lambda^{2}}\text{tan}(\frac{1}{2}\left(c+\xi \right)\sqrt{4\mu -\lambda^{2}})}\\ & -\frac{120\mu^{2}}{{\left(\lambda -\sqrt{4\mu -\lambda^{2}}\text{tan}(\frac{1}{2}\left(c+\xi \right)\sqrt{4\mu -\lambda^{2}})\right)}^{2}}. \end{array} \end{array}$$ (20)

**Fig 2:**
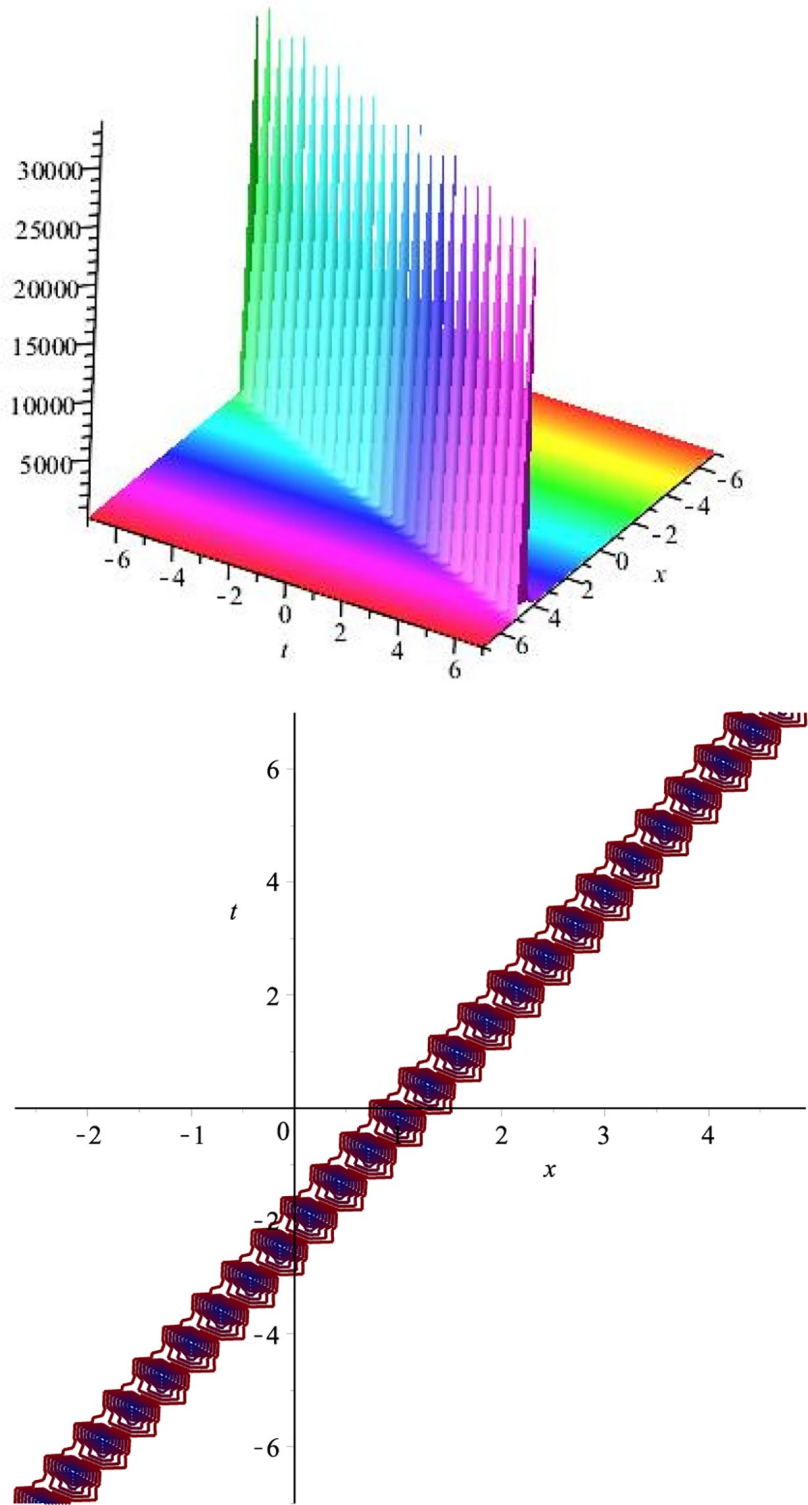
Singular soliton: ∣*v*_2_(*x*, *t*)∣ for *l* = 0.67, *m* = 2.1, λ = 1.5, *c* = −1.4, *v* = 0.5, *μ* = 2.5.

The graphical representation of Eq (20) is displayed in Fig 2.

**Case 3**: λ^2^ − 4*μ* > 0, *μ* = 0 **and** λ ≠ 0

Case 3 gives hyperbolic solution, as

$$\begin{array}{c} \begin{array}{cc} v_{3}\left(x,t\right)= & -\frac{\sqrt{3}\sqrt{3l^{2}-10\lambda^{2}lm+35\lambda^{4}m^{2}}+3l}{4m}\\ & \frac{1}{8}\left(-15\right)(\lambda^{2}\text{cosh}\left(\lambda \left(c+\xi \right)\right)+3\lambda^{2}){\text{csch}}^{2}(\frac{1}{2}\lambda \left(c+\xi \right)). \end{array} \end{array}$$ (21)

**Case 4**: λ^2^ − 4*μ* = 0, *μ* ≠ 0 **and** λ ≠ 0

Case 4 give rational solution, as

$$\begin{array}{c} \begin{array}{cc} v_{4}\left(x,t\right)= & -\frac{3\sqrt{l^{2}}+3l+15m(\lambda^{2}+4\mu )}{4m}+\frac{15\lambda^{3}\left(c+\xi \right)}{c\lambda +\lambda \xi -1}-\frac{30\lambda^{4}{\left(c+\xi \right)}^{2}}{{\left(2-2\lambda \left(c+\xi \right)\right)}^{2}}. \end{array} \end{array}$$ (22)

**Case 5**: λ^2^ − 4*μ* = 0, *μ* = 0 **and** λ = 0

Applying the condition of Case 5, the following solution is obtained, as

$$\begin{array}{c} \begin{array}{cc} v_{5}\left(x,t\right)= & \{-\frac{30}{{\left(c+\xi \right)}^{2}}-\frac{3\sqrt{l^{2}}+3l}{4m}\}. \end{array} \end{array}$$ (23)

2^*nd*^ **Solution set**:

$$\begin{array}{ccc} & & w_{0}=-30\mu ,\;w_{1}=-30\lambda ,\;w_{2}=-30,\;\beta =1. \end{array}$$

By inserting the values of 2^*nd*^ **Solution set** in Eq (18), the following solutions are obtained.

**Case 1**: λ^2^ − 4*μ* > 0, *μ* ≠ 0

In this case hyperbolic solution is obtained, as

$$\begin{array}{c} \begin{array}{cc} v_{6}\left(x,t\right)= & -30\mu -\frac{60\lambda \mu }{-\sqrt{\lambda^{2}-4\mu }\;\text{tanh}\;(\frac{1}{2}\left(c+\xi \right)\sqrt{\lambda^{2}-4\mu })-\lambda }\\ & -\frac{60\mu }{-\sqrt{\lambda^{2}-4\mu }\;\text{tanh}\;(\frac{1}{2}\left(c+\xi \right)\sqrt{\lambda^{2}-4\mu })-\lambda }. \end{array} \end{array}$$ (24)

**Fig 3:**
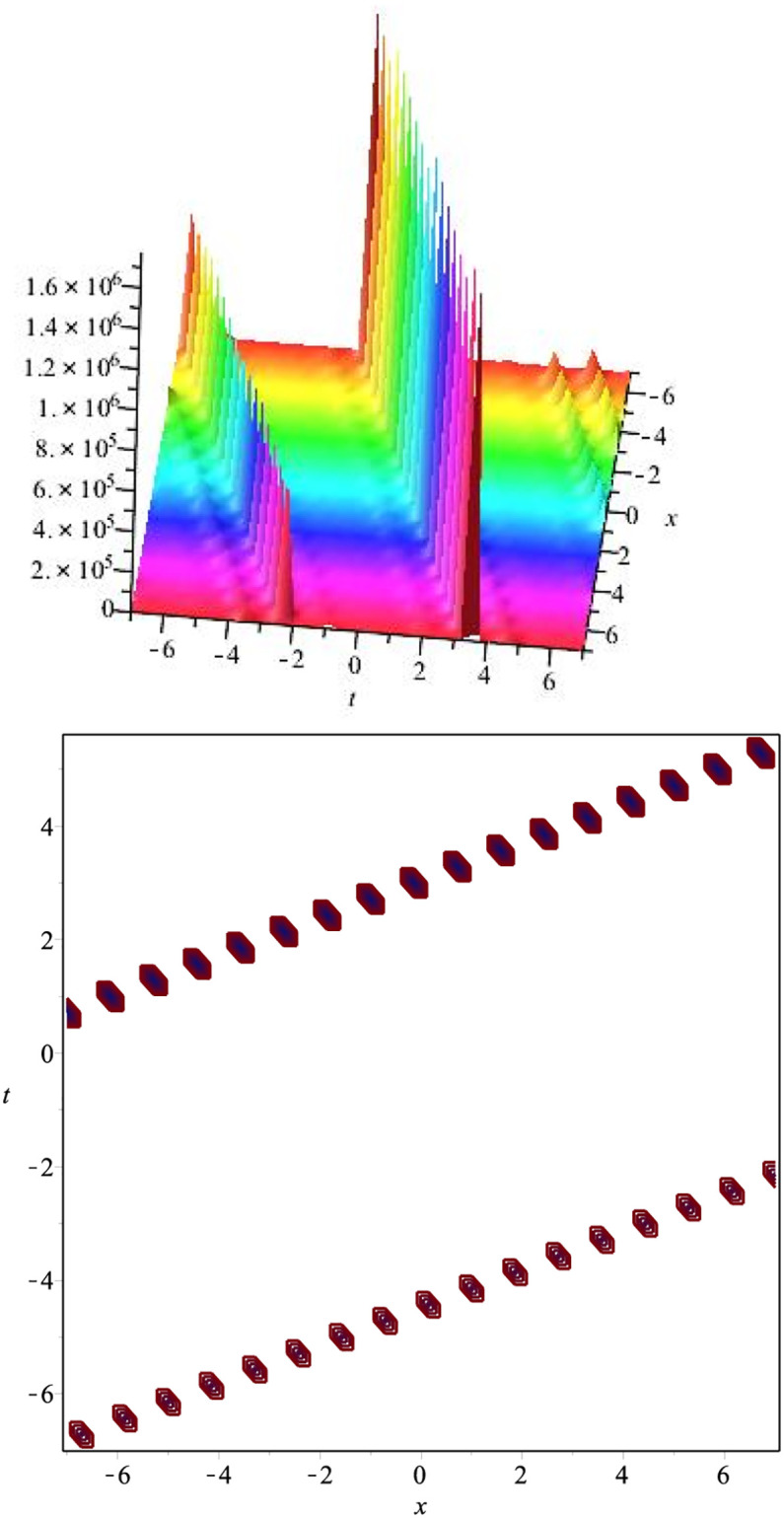
Singular soliton: ∣*v*_6_(*x*, *t*)∣ for *l* = 3.1, *m* = 4.2, λ = 1.8, *c* = 3.8, *v* = 3, *μ* = 3.6.

The graphical representation of *v*_6_(*x*, *t*) is presented in Fig 3.

**Case 2**: λ^2^ − 4*μ* < 0, *μ* ≠ 0

Trigonometric function solution has been obtained in this case, as

$$\begin{array}{c} \begin{array}{cc} v_{7}\left(x,t\right)= & -30\mu -\frac{120\mu^{2}}{{\left(\sqrt{4\mu -\lambda^{2}}\;\text{tan}\;(\frac{1}{2}\left(c+\xi \right)\sqrt{4\mu -\lambda^{2}})-\lambda \right)}^{2}}\\ & -\frac{60\lambda \mu }{\sqrt{4\mu -\lambda^{2}}\text{tan}(\frac{1}{2}\left(c+\xi \right)\sqrt{4\mu -\lambda^{2}})-\lambda }. \end{array} \end{array}$$ (25)

**Case 3**: λ^2^ − 4*μ* > 0, *μ* = 0 **and** λ ≠ 0

Hyperbolic function solution is obtained, as

$$\begin{array}{c} \begin{array}{cc} v_{8}\left(x,t\right)= & \frac{w_{2}(l+5\lambda^{2}m)}{20m}+\frac{\lambda^{2}w_{2}}{{\left(\text{sinh}\left(\lambda \left(c+\xi \right)\right)+\text{cosh}\left(\lambda \left(c+\xi \right)\right)-1\right)}^{2}}\\ & +\frac{\lambda^{2}w_{2}}{\text{sinh}(\lambda (c+\xi ))+\text{cosh}(\lambda (c+\xi ))-1}. \end{array} \end{array}$$ (26)

**Case 4**: λ^2^ − 4*μ* = 0, *μ* ≠ 0 **and** λ ≠ 0

Rational solution has been obtained, as

$$\begin{array}{c} v_{9}\left(x,t\right)=-30\mu -\frac{30\lambda^{4}{\left(c+\xi \right)}^{2}}{{\left(2-2\lambda \left(c+\xi \right)\right)}^{2}}-\frac{30\lambda^{3}\left(c+\xi \right)}{2-2\lambda (c+\xi )}. \end{array}$$ (27)

**Case 5**: λ^2^ − 4*μ* = 0, *μ* = 0 **and** λ = 0

Following solution has been obtained, as

$$\begin{array}{c} v_{10}\left(x,t\right)=-\frac{30}{{\left(c+\xi \right)}^{2}}. \end{array}$$ (28)

3^*rd*^ **Solution set**:

$$\begin{array}{ccc} & & w_{0}=\frac{w_{2}(l+5\lambda^{2}m)}{20m},\;w_{1}=\lambda w_{2},\;\mu =\frac{l+5\lambda^{2}m}{20m},\;\beta =-\frac{1800}{w_{2}(w_{2}+90)},\;\rho =-\frac{4l^{2}}{25m}. \end{array}$$

By putting the above values of 3^*rd*^ **Solution set** different solutions has been obtained as follows:

**Case 1**: λ^2^ − 4*μ* > 0, *μ* ≠ 0

In this case hyperbolic solution is extracted, as

$$\begin{array}{c} v_{11}\left(x,t\right)=\frac{lw_{2}(l+5\lambda^{2}m)}{4m{\left(\sqrt{5}\sqrt{l}\;\text{sinh}\;(\frac{\sqrt{l}\left(c+\xi \right)}{2\sqrt{5}\sqrt{m}})+5\lambda \sqrt{m}\;\text{cosh}\;(\frac{\sqrt{l}\left(c+\xi \right)}{2\sqrt{5}\sqrt{m}})\right)}^{2}}, \end{array}$$ (29)

provided that *lm* < 0.

**Case 2**: λ^2^ − 4*μ* < 0, *μ* ≠ 0

Trigonometric function solution is obtained, as

$$\begin{array}{c} v_{12}\left(x,t\right)=\frac{lw_{2}(l+5\lambda^{2}m)}{4m{\left(\sqrt{5}\sqrt{l}\;\text{sin}\;(\frac{\sqrt{l}\left(c+\xi \right)}{2\sqrt{5}\sqrt{m}})-5\lambda \sqrt{m}\;\text{cos}\;(\frac{\sqrt{l}\left(c+\xi \right)}{2\sqrt{5}\sqrt{m}})\right)}^{2}}, \end{array}$$ (30)

provided that *lm* > 0.

**Case 3**: λ^2^ − 4*μ* > 0, *μ* = 0 **and** λ ≠ 0

Hyperbolic solution is presented in this case.

$$\begin{array}{c} \begin{array}{cc} v_{13}\left(x,t\right)= & \frac{w_{2}(l+5\lambda^{2}m)}{20m}\frac{\lambda^{2}w_{2}}{\text{sinh}(\lambda (c+\xi ))+\text{cosh}(\lambda (c+\xi ))-1}\\ & +\frac{\lambda^{2}w_{2}}{{\left(\text{sinh}\left(\lambda \left(c+\xi \right)\right)+\text{cosh}\left(\lambda \left(c+\xi \right)\right)-1\right)}^{2}}. \end{array} \end{array}$$

**Fig 4:**
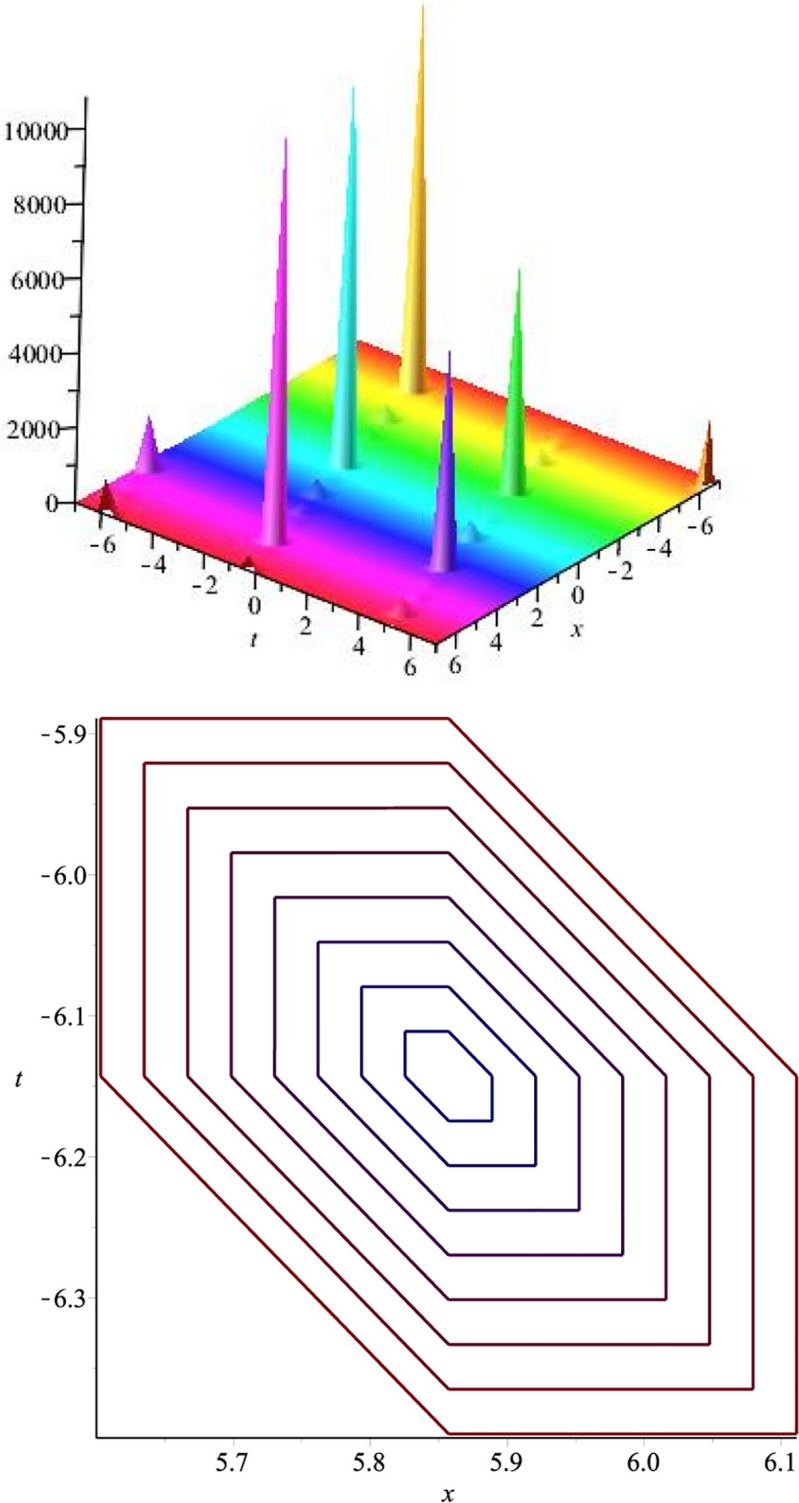
Singular soliton: ∣*v*_13_(*x*, *t*)∣ for *l* = −0.9, *m* = −1.8, λ = −2.3, *c* = 2.7, *v* = 3.6, *μ* = 2.3.

The graphical representation of the above constructed solution is represented in Fig 4.

**Case 4**: λ^2^ − 4*μ* = 0, *μ* ≠ 0 **and** λ ≠ 0

Rational form of solution is obtained, as

$$\begin{array}{c} v_{14}\left(x,t\right)=\frac{1}{20}w_{2}(\frac{5\lambda^{2}}{{\left(\lambda \left(c+\xi \right)-1\right)}^{2}}+\frac{l}{m}). \end{array}$$ (31)

**Case 5**: λ^2^ − 4*μ* = 0, *μ* = 0 **and** λ = 0

Following solution for Case 5 has been obtained, as

$$\begin{array}{c} v_{15}\left(x,t\right)=\frac{1}{20}w_{2}(\frac{5{\left(\lambda \left(c+\xi \right)+2\right)}^{2}}{{\left(c+\xi \right)}^{2}}+\frac{l}{m}), \end{array}$$ (32)

where *c* is constant of integration.

### Construction of soliton solutions via technique II

In this subsection, the technique II has been applied on the proposed model to obtain new soliton solutions. Applying homogenous balancing on Eq (17) yields *n* = 2. For *n* = 2, Eq (11) becomes

$$\begin{array}{c} s\left(\xi \right)=c_{0}+c_{1}(\frac{G^{{}^{\prime }}}{G^{2}})+c_{2}(\frac{G^{{}^{\prime }}}{G^{2}}{)}^{2}+d_{1}(\frac{G^{{}^{\prime }}}{G^{2}}{)}^{-1}+d_{2}(\frac{G^{{}^{\prime }}}{G^{2}}{)}^{-2}. \end{array}$$ (33)

Utilizing technique II, following solution sets have been derived.

**Solution set 1**:

$$\begin{array}{cc} & c_{0}=4(4\sqrt{30}-15)\lambda \mu ,\;c_{1}=0,\;d_{1}=0,\;c_{2}=-30\lambda^{2},\;d_{2}=-30\mu^{2},\\ & \rho =\frac{16}{5}(2+\sqrt{30})\lambda l\mu ,\;m=\frac{l}{80\lambda \mu },\;\beta =1. \end{array}$$ (34)

Upon inserting these values in Eq (33), the following solutions are obtained. λ and *μ* are considered as arbitrary constants.

**Case 1**: λ*μ* > 0

In this particular case trigonometric function solution is obtained, as

$$\begin{array}{c} \begin{array}{cc} v_{16}\left(x,t\right)= & 4(4\sqrt{30}-15)\lambda \mu -\frac{30\lambda \mu (H_{2}\;\text{cos}\;(\xi \sqrt{\lambda \mu })-H_{1}\;\text{sin}\;(\xi \sqrt{\lambda \mu }){)}^{2}}{{\left(H_{1}\;\text{cos}\;(\xi \sqrt{\lambda \mu })+H_{2}\;\text{sin}\;(\xi \sqrt{\lambda \mu })\right)}^{2}}\\ & -\frac{30\lambda \mu {\left(H_{1}\;\text{cos}\;(\xi \sqrt{\lambda \mu })+H_{2}\;\text{sin}\;(\xi \sqrt{\lambda \mu })\right)}^{2}}{{\left(H_{2}\;\text{cos}\;(\xi \sqrt{\lambda \mu })-H_{1}\;\text{sin}\;(\xi \sqrt{\lambda \mu })\right)}^{2}}. \end{array} \end{array}$$ (35)

**Case 2**: λ*μ* < 0

In this case hyperbolic function solution is obtained, as

$$\begin{array}{c} \begin{array}{cc} v_{17}\left(x,t\right)= & 4(4\sqrt{30}-15)\lambda \mu -\frac{30\lambda \mu^{2}(H_{1}\;\text{sinh}\;(2\xi \sqrt{\left|\lambda \mu \right|})+H_{1}\;\text{cosh}\;(H_{2}-2\xi \sqrt{\left|\lambda \mu \right|})){}^{2}}{|\lambda \mu |(H_{1}\;\text{sinh}\;(2\xi \sqrt{\left|\lambda \mu \right|})+H_{1}\;\text{cosh}\;(2\xi \sqrt{\left|\lambda \mu \right|}+H_{2})){}^{2}}\\ & -\frac{30\lambda |\lambda \mu |(H_{1}\;\text{sinh}\;(2\xi \sqrt{\left|\lambda \mu \right|})+H_{1}\;\text{cosh}\;(2\xi \sqrt{\left|\lambda \mu \right|}+H_{2})){}^{2}}{(H_{1}\;\text{sinh}\;(2\xi \sqrt{\left|\lambda \mu \right|})+H_{1}\;\text{cosh}\;(H_{2}-2\xi \sqrt{\left|\lambda \mu \right|})){}^{2}}. \end{array} \end{array}$$ (36)

**Case 3**: λ = 0 **and** *μ* ≠ 0

In this case rational form of solution is obtained, as

$$\begin{array}{c} \begin{array}{c} v_{18}\left(x,t\right)-\frac{30\mu^{4}(H_{1}\xi +H_{2}){}^{2}}{H_{1}^{2}}. \end{array} \end{array}$$ (37)

**Solution set 2**:

$$\begin{array}{ccc} & & c_{0}=-\frac{\sqrt{3}\sqrt{3l^{2}+40\lambda \mu lm+560\lambda^{2}\mu^{2}m^{2}}+3l+60\lambda \mu m}{4m},\;c_{1}=0,\;d_{1}=0,\\ & & c_{2}=-30\lambda^{2},\;d_{2}=0,\;\beta =1. \end{array}$$

Taking values of **Solution set 2** in Eq (33) solutions are of the following form:

**Case 1**: λ*μ* > 0

Through this case trigonometric function solution is extracted, as

$$\begin{array}{ccc} v_{19}\left(x,t\right) & = & -\frac{\sqrt{3}\sqrt{3l^{2}+40\lambda \mu lm+560\lambda^{2}\mu^{2}m^{2}}+3l+60\lambda \mu m}{4m}\\ & & -\frac{30\lambda \mu (H_{2}\;\text{sin}\;(\xi \sqrt{\lambda \mu })+H_{1}\;\text{cos}\;(\xi \sqrt{\lambda \mu })){}^{2}}{(H_{2}\;\text{cos}\;(\xi \sqrt{\lambda \mu })-H_{1}\;\text{sin}\;(\xi \sqrt{\lambda \mu })){}^{2}}. \end{array}$$ (38)

**Case 2**: λ*μ* < 0

In this case hyperbolic function solution is obtained, as

$$\begin{array}{c} \begin{array}{cc} v_{20}\left(x,t\right)= & -\frac{\sqrt{3}\sqrt{3l^{2}+40\lambda \mu lm+560\lambda^{2}\mu^{2}m^{2}}+3l+60\lambda \mu m}{4m}\\ & -\frac{30\lambda |\lambda \mu |(H_{1}\;\text{sinh}\;(2\xi \sqrt{\left|\lambda \mu \right|})+H_{1}\;\text{cosh}\;(2\xi \sqrt{\left|\lambda \mu \right|}+H_{2})){}^{2}}{(H_{1}\;\text{sinh}\;(2\xi \sqrt{\left|\lambda \mu \right|})+H_{1}\;\text{cosh}\;(2\xi \sqrt{\left|\lambda \mu \right|}-H_{2})){}^{2}}. \end{array} \end{array}$$ (39)

**Fig 5:**
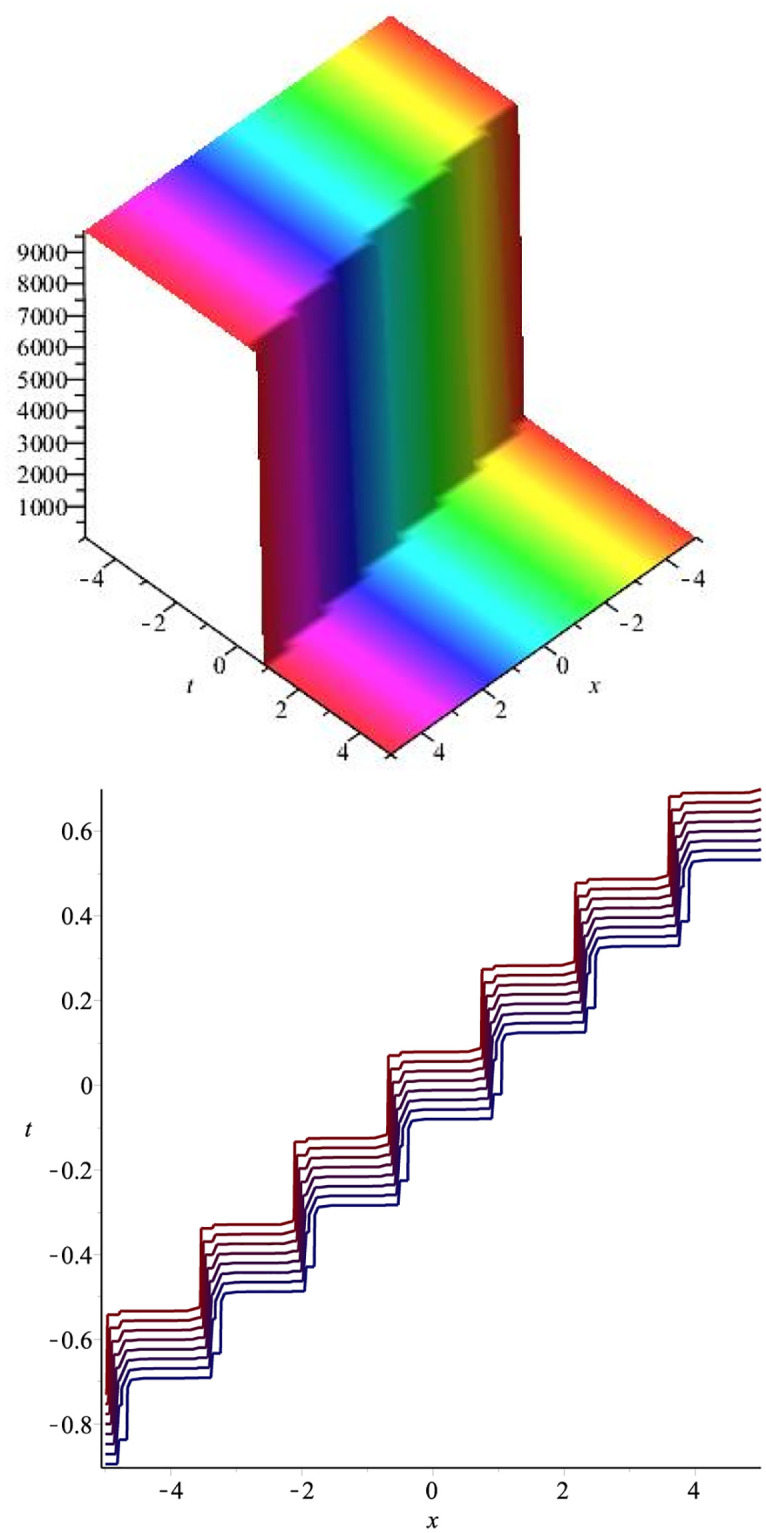
Kink soliton: ∣*v*_20_(*x*, *t*)∣ for *β* = 2.5, *v* = 7, λ = 3.7, *μ* = −3.2, *H*_1_ = 1.5, *H*_1_ = 1, *l* = 1.3, *m* = 2.6.

The graphical representation of the above hyperbolic solution is plotted in Fig 5.

**Case 3**: λ = 0 **and** *μ* ≠ 0

In this case rational form of solution is obtained, as

$$\begin{array}{c} -\frac{3\sqrt{l^{2}}+3l}{4m}. \end{array}$$ (40)

**Solution set 3**:

$$\begin{array}{cc} & c_{0}=-\frac{30\mu (\sqrt{\beta \left(9\beta -8\right)\lambda^{4}}+3\beta \lambda^{2})}{\beta \lambda },\;c_{1}=0,\;d_{1}=0,\;c_{2}=-\frac{15(\sqrt{\beta \left(9\beta -8\right)\lambda^{4}}+3\beta \lambda^{2})}{\beta },\\ & d_{2}=-\frac{15\mu^{2}(\sqrt{\beta \left(9\beta -8\right)\lambda^{4}}+3\beta \lambda^{2})}{\beta \lambda^{2}},\;\rho =\frac{1}{5}\left(-64\right)\lambda l\mu ,\;m=\frac{l}{80\lambda \mu }. \end{array}$$

Inserting parameters of **Solution set 3** in Eq (33), the solutions obtained are as follows:

**Case 1**: λ*μ* > 0

Here trigonometric function solution has been obtained, as

$$\begin{array}{c} \begin{array}{cc} v_{22}\left(x,t\right)= & -\frac{15\mu (\sqrt{\beta \left(9\beta -8\right)\lambda^{4}}+3\beta \lambda^{2})(H_{2}\;\text{sin}\;(\xi \sqrt{\lambda \mu })+H_{1}\;\text{cos}\;(\xi \sqrt{\lambda \mu })){}^{2}}{\beta \lambda (H_{2}\;\text{cos}\;(\xi \sqrt{\lambda \mu })-H_{1}\;\text{sin}\;(\xi \sqrt{\lambda \mu })){}^{2}}\\ & -\frac{15\mu (\sqrt{\beta \left(9\beta -8\right)\lambda^{4}}+3\beta \lambda^{2})(H_{2}\;\text{cos}\;(\xi \sqrt{\lambda \mu })-H_{1}\;\text{sin}\;(\xi \sqrt{\lambda \mu })){}^{2}}{\beta \lambda (H_{2}\;\text{sin}\;(\xi \sqrt{\lambda \mu })+H_{1}\;\text{cos}\;(\xi \sqrt{\lambda \mu })){}^{2}}\\ & -\frac{30\mu (\sqrt{\beta \left(9\beta -8\right)\lambda^{4}}+3\beta \lambda^{2})}{\beta \lambda }. \end{array} \end{array}$$

**Case 2**: λ*μ* < 0

Hyperbolic function solution has been obtained, as

$$\begin{array}{c} \begin{array}{cc} v_{23}\left(x,t\right)= & -\frac{15\mu^{2}(\sqrt{\beta \left(9\beta -8\right)\lambda^{4}}+3\beta \lambda^{2})(H_{1}\;\text{sinh}\;(2\xi \sqrt{\left|\lambda \mu \right|})+H_{1}\;\text{cosh}\;(2\xi \sqrt{\left|\lambda \mu \right|}-H_{2})){}^{2}}{\beta \lambda |\lambda \mu |(H_{1}\;\text{sinh}\;(2\xi \sqrt{\left|\lambda \mu \right|})+H_{1}\;\text{cosh}\;(2\xi \sqrt{\left|\lambda \mu \right|}+H_{2})){}^{2}}\\ & -\frac{15(\sqrt{\beta \left(9\beta -8\right)\lambda^{4}}+3\beta \lambda^{2})|\lambda \mu |(H_{1}\;\text{sinh}\;(2\xi \sqrt{\left|\lambda \mu \right|})+H_{1}\;\text{cosh}\;(2\xi \sqrt{\left|\lambda \mu \right|}+H_{2})){}^{2}}{\beta \lambda (H_{1}\;\text{sinh}\;(2\xi \sqrt{\left|\lambda \mu \right|})+H_{1}\;\text{cosh}\;(2\xi \sqrt{\left|\lambda \mu \right|}-H_{2})){}^{2}}\\ & -\frac{30\mu (\sqrt{\beta \left(9\beta -8\right)\lambda^{4}}+3\beta \lambda^{2})}{\beta \lambda }, \end{array} \end{array}$$ (41)

where *H*_1_ and *H*_2_ are considered as arbitrary constants.

## Graphical overview of selected solution sets

Graphical presentations of a few of the retrieved exact soliton solutions of the KdV-CDG equation are discussed in this section. Using a computer simulated program Maple, different 3D and contour plots have been plotted. The most appropriate values of arbitrary parameters have been chosen to construct 3D surface plots and 2D graphs.

## Conclusion

In this article, two unique and reliable techniques, the extended $\frac{G^{\prime }}{G^{2}}$-expansion method and exponential (*ψ*(*ξ*))-expansion method have been employed for constructing the exact solutions of the Korteweg-de Vries Caudrey-Dodd-Gibbon (KdV-CDG) equation. These technique efficiently extracted hyperbolic, rational and trigonometric solutions. These solutions can be found useful in investigating the governing model in different fields of science and engineering. 3D surface plots and 2D graphs have also been represented in this article to describe the dynamics of the obtained solutions.

## References

[1] Zhang LD, Tian SF, Zhang TT, Yan XJ. Characteristics of solitary waves, breather waves and hybrid waves to a new (3 + 1)-dimensional nonlinear evolution equation in a quantum magnetoplasma. EPL. 2021;135(2):20003. doi: 10.1209/0295-5075/135/20003

[2] Li ZQ, Tian SF, Peng WQ, Yang JJ. Inverse scattering transform and soliton classification of higher-order nonlinear Schrödinger-Maxwell-Bloch equations. Theor Math Phys. 2020;203(3):709–725. doi: 10.1134/S004057792006001X

[3] Tian SF, Wang XF, Zhang TT, Qiu WH. Stability analysis, solitary wave and explicit power series solutions of a (2 + 1)-dimensional nonlinear Schrödinger equation in a multicomponent plasma. INT J Numer Method H. 2021;3(5):1732–1748. doi: 10.1108/HFF-08-2020-0517

[4] Yang JJ, Tian SF, Li ZQ. Riemann-Hilbert problem for the focusing nonlinear Schrödinger equation with multiple high-order poles under nonzero boundary conditions. Phys D: Nonlinear Phenom. 2022;432:133162. doi: 10.1016/j.physd.2022.133162

[5] Yan XW, Tian SF, Dong MJ, Zhang TT. Rogue Waves and Their Dynamics on Bright-Dark Soliton Background of the Coupled Higher Order Nonlinear Schrödinger Equation. J Phys Soc Japan. 2019;88(7):074004. doi: 10.7566/JPSJ.88.074004

[6] Yin ZY, Tian SF. Nonlinear wave transitions and their mechanisms of (2 + 1)-dimensional Sawada-Kotera equation. Phys D: Nonlinear Phenom. 2021;427:133002. doi: 10.1016/j.physd.2021.133002

[7] Akram G, Arshed S, Sadaf M, Sameen F. The generalized projective Riccati equations method for solving quadratic-cubic conformable time-fractional Klien-Fock-Gordon equation. Ain Shams Eng J. 2021;13:101658.

[8] Akram G, Sadaf M, Dawood M, Baleanu D. Optical solitons for Lakshmanan-Porsezian-Daniel equation with Kerr law non-linearity using improved tan(*ψ*(*η*)/2)-expansion technique. Results Phys. 2021;29:104758. doi: 10.1016/j.rinp.2021.104758

[9] Duran S. Extractions of travelling wave solutions of (2+ 1)-dimensional Boiti-Leon-Pempinelli system via $\left(\frac{G^{\prime }}{G},\frac{1}{G}\right)$-expansion method. Opt Quantum Electron. 2021;53:299. doi: 10.1007/s11082-021-02940-w

[10] Cheng J, Tian SF, Wu ZJ. On the $\bar{\partial }$-problem and dressing method for the complex vector modified KdV equation. Theor Math Phys. 2021;209(2):305–326. doi: 10.1134/S0040577921110064

[11] Wang ZY, Tian SF, Cheng J. The $\bar{\partial }$-dressing method and soliton solutions for the three-component coupled Hirota equations. J Math Phys. 2021;62(9):093510. doi: 10.1063/5.0046806

[12] Arshed S, Sadia M. $\frac{G^{\prime }}{G^{2}}$-expansion method: New traveling wave solutions for some nonlinear fractional partial differential equations. Opt Quantum Electron. 2018;50:123. doi: 10.1007/s11082-018-1391-6

[13] Akram G, Gillani SR. Sub pico-second Soliton with Triki-Biswas equation by the extended $\frac{G^{\prime }}{G^{2}}$-expansion method and the modified auxiliary equation method. Optik. 2021;229:166227. doi: 10.1016/j.ijleo.2020.166227

[14] Yang J, Feng Q. Using the improved (*φ*(*ξ*))-expansion method to find the soliton solutions of the nonlinear evolution equation. Eur Phys J Plus. 2021;136:348. doi: 10.1140/epjp/s13360-021-01321-2

[15] Kumar S, Kumar A, Wazwaz AM. New exact solitary wave solutions of the strain wave equation in microstructured solids via the generalized exponential rational function method. Eur Phys J Plus. 2020;135:870. doi: 10.1140/epjp/s13360-020-00883-x

[16] Akbar MA, Ali NHM, Tanjim T. Adequate soliton solutions to the perturbed Boussinesq equation and the KdV-Caudrey-Dodd-Gibbon equation. J King Saud Univ Sci. 2020;32(6):2777–2785. doi: 10.1016/j.jksus.2020.06.014

[17] Dai CQ, Wang YY, Fan Y, Zhang JF. Interactions between exotic multi-valued solitons of the (2+ 1)-dimensional Korteweg-de Vries equation describing shallow water wave. Appl Math Model. 2020;80:506–515. doi: 10.1016/j.apm.2019.11.056

[18] Yaslan HC, Girgin A. New exact solutions for the conformable space-time fractional KdV, CDG, (2 + 1)-dimensional CBS and (2 + 1)-dimensional AKNS equations. J Taibah Univ Sci. 2019;13(1):1–8. doi: 10.1080/16583655.2018.1515303

[19] Wazwaz AM. N-soliton solution for the combined KdV-CDG equation and the KdV-Lax equation. Appl Math Comput. 2008;203(1):402–407.

[20] Biswas A, Ebadi G, Triki H, Yildirim A, Yousefzadeh N. Topological soliton and other exact solutions to KdV-Caudrey-Dodd-Gibbon equation. Results Math. 2013;63(1):687–703. doi: 10.1007/s00025-011-0226-6

[21] Ma H, Huang H, Deng A. Soliton molecules, asymmetric solitons and hybrid solutions for KdV-CDG equation. Partial Differential Equations in Applied Mathematics. 2021;5:100214. doi: 10.1016/j.padiff.2021.100214

[22] Asjad MI, Rehman HU, Ishfaq Z, Awrejcewicz J, Akgül A, Riaz MB. On Soliton Solutions of Perturbed Boussinesq and KdV-Caudery-Dodd-Gibbon Equations. Coatings. 2021;11(11):1429. doi: 10.3390/coatings11111429

